# Editorial: Autoimmune Diabetes: Molecular Mechanisms and Neoantigens

**DOI:** 10.3389/fimmu.2022.864517

**Published:** 2022-02-18

**Authors:** Sally C. Kent, Arnaud Zaldumbide

**Affiliations:** ^1^ Diabetes Center of Excellence, Department of Medicine, University of Massachusetts Chan Medical School, Worcester, MA, United States; ^2^ Department of Cell and Chemical Biology, Leiden University Medical Center, Leiden, Netherlands

**Keywords:** type 1 diabetes, neoantigens, beta cell (β cell), autoimmunity, immunotherapy, T cells, stress

Hundred years after the discovery of insulin by Banting, Best, and McLeod that fostered hope across type 1 diabetic population, there is still no cure for type 1 diabetes (T1D). Perhaps worse, the molecular mechanisms involved in the destruction of insulin producing cells in the pancreas remain poorly understood hampering the development of novel therapeutic strategies. While the scenario is clear and characterized by the selective and progressive destruction of the insulin-producing pancreatic beta cells by the invading immune cells, our view on the participation of the different actors has evolved over the years. From the view that T1D is a solely T cell mediated disorder, in which an impaired thymic education or the participation of low affinity T-cells were believed to be responsible for the immune attack, increasing evidence has positioned the beta cell as main player in their own destruction with physiological/metabolic stress on the beta cell as a major contributor to beta cell dysfunction The increased splicing events in beta cells when maintained under pathophysiological conditions and the T-cell autoreactivity detected against defective ribosomal products, deamidated/citrullinated autoantigens or fusion epitopes illustrate that transcriptional, translational, post-transcriptional and post-translational processes can be affected during insulitis and support the participation of neoantigens in T1D pathology and position the beta cell as the main actor in their destruction process. In addition, the roles of multiple islet-infiltrating immune cell subpopulations in the pathophysiology of beta cell dysregulation and death are now coming into focus. While the development of therapies targeting beta cell dysfunction/destruction and the autoimmune attack for T1D remains challenging, this increased understanding of these processes in individuals at-risk for or with T1D is beneficial.

In this Research Topic, Rodriguez-Calvo et al. propose an overview of the different causes and molecular mechanisms involved in the generation of these neoepitopes and speculate on how those non-native proteins could drive autoimmunity and precipitate beta cell destruction. The evaluation of their role as biomarkers for disease progression and target for immune intervention is also discussed. Among the different processes that participate to the generation of neoantigens, many studies have investigated the impact of citrullinated proteins to the development of autoimmunity. Yang et al. detail the role of calcium-dependent peptidylarginine deiminase (PAD) enzymes in the generation of citrullinated proteins and their impact on the antigenicity of beta cell antigens, echoing studies on other autoimmune diseases (e.g. rheumatoid arthritis (RA), systemic lupus erythematosus (SLE), multiple sclerosis (MS), psoriasis, Sjögren’s syndrome (SS), anti-phospholipid syndrome (ALS) and inflammatory bowel disease (IBD) in which citrullination of self-protein has been implicated). Building on convincing data from NOD mice, they show the potential of the chemical inhibition of PAD enzyme in preventing and delaying T1D incidence, the authors elaborate on the potential of PAD inhibitor as therapeutic strategy for new onset T1D patients. Furthermore, while You et al. are questioning the role of the microbiome composition in triggering autoimmunity exploring, in NOD mice models, the participation of gut leakage and neutrophil extracellular traps (NETs) in T1D progression, they identified PAD4 as important player during NETosis, providing additional proofs for therapeutic approach targeting PAD enzymes.

Also in this Research Topic, Mannering et al. reviewed the historical discovery of T1D associated hybrid insulin peptides (HIPs) and discussed the mechanisms leading to the formation of non-contiguous peptides formed by cis/trans peptidation or reverse proteolysis during protein processing and degradation in crinosomes. Despite the large number of possible combinations and the low concentration of HIPs in beta cells that render their detection challenging by peptidomics analyses, Wiles et al. identified a novel non-contiguous C-peptide epitope (insC_4-26_ – insC_1-3_) in human islets and measure a CD4 peptide response in T1D patients by tetramer staining illustrating the important role of HIP peptides in T cell immunity to beta cells.

The concept of peptide fusion significantly expands the peptidome diversity that may participate to the break in tolerance observed in T1D. Revisiting the molecular mimicry hypothesis, Mishto et al. assess whether cis peptides derived viruses could trigger T-cell mediated autoimmunity. Using in silico prediction they compared zwitter cis spliced peptides with sequences from medullary thymic epithelial cells (mTEC)and beta cells and identify potential diabetogenic viral-human hybrid antigens.

In this issue, Arif et al. propose an elegant combinatorial approach (phenotypic, transcriptomic and clonotypic analysis) to evaluate the peripheral T cell responses to native versus PTM dependent auto antigenic peptides in T1D and understand the break in peripheral tolerance to beta cell antigens. Despite an evident T cell response mounted against beta cells, Rodriguez-Calvo et al. questioned the participation of non-islet reactive T cells to T1D and discuss a role as “partners in crime” in the constitution of the inflamed islets’ microenvironment.

An important mechanism by which cytokines contribute to beta cell death and amplification of the inflammation is *via* induction of ER stress and subsequent unfolded protein response pathway. Like the endoplasmic reticulum, mitochondria are complex and dynamic organelles that play a key role in beta cell function by coupling glucose metabolism to insulin secretion and regulating apoptotic cell death. As described by Vig et al. the interaction between the ER and mitochondria during the adaptive mechanism to environmental stress indicate that both organelles orchestrate the communication between the beta cells and the immune system compartment. Prolonged exposure to metabolic or inflammatory stress promotes additional coping mechanisms including initiation of recycling programs and selective secretion of proteins and small RNA in microvesicles. In this Research Topic, Grieco et al. discuss the biogenesis and mechanisms of action of these extracellular vesicles and examine their role as mediator between the beta cells and the immune cells in particular as primary event in activating APCs. In addition, Leenders et al. describe another aspect of the stress response demonstrating a role of oxidative stress in the change of beta cell identity. Using primary human islets, they show that hydrogen peroxide triggers a beta cell dysfunction associated with a loss of beta cell specific markers.

Altogether the different studies presented in this issue, provide an update on the latest developments in fundamental and clinical T1D research. Yet, as conclusion, Thomas et al. identify the remaining challenges, focusing on antigen specific immunotherapy, emphasizing the hurdles encountered in the bench to bed side transition but also the novel opportunities from T1D consortia that facilitate the identification of subjects who could benefit from such immunotherapies.

## Author Contributions

SCK and AZ act as guest editor of this issue and wrote the editorial. Both authors contributed to the article and approved the submitted version.

## Conflict of Interest

The authors declare that the research was conducted in the absence of any commercial or financial relationships that could be construed as a potential conflict of interest.

## Publisher’s Note

All claims expressed in this article are solely those of the authors and do not necessarily represent those of their affiliated organizations, or those of the publisher, the editors and the reviewers. Any product that may be evaluated in this article, or claim that may be made by its manufacturer, is not guaranteed or endorsed by the publisher.

